# Supplementary material for: Krüppel-homologue 1 Mediates Hormonally Regulated Dominance Rank in a Social Bee
Source: Biology (Basel). 2021 Nov 15;10(11):1188. doi: 10.3390/biology10111188 (PMC8614866; doi:10.3390/biology10111188)
Supplement: Supplementary file 1 [file biology-10-01188-s001.zip › biology-1401796-supplementary.pdf]

Supplementary Information

Krüppel-homologue 1 mediates hormonally-regulated dominance rank in a social bee

Atul Pandey[1,2*] and Guy Bloch[1*]

[1]Department of Ecology, Evolution and Behavior, The Hebrew University of Jerusalem, Jerusalem, Israel; guy.bloch@mail.huji.ac.il

[2]Current address: Department of Ecology and Evolutionary Biology, University of Michigan, Ann Arbor, Michigan, USA; atulkp@umich.edu
* Correspondence: atulkp@umich.edu (A.P), guy.bloch@mail.huji.ac.il (G.B)

Supplementary table S1. List of primers used in the study.

	Primers	Direction	Sequence	Amplicon Length
1	Krupple homolog (*Kr-h1*)-long	Forward	TTGCATCAGGTTGCCCACTA	668
		Reverse	CACCGTTTTCTTGGAGGGAGA	
2	*Kr-h1*–T7 nested	Forward	TAATACGACTCACTATAGGGAGAAAAACTCATTCAGACTCATCGGT	440
		Reverse	TAATACGACTCACTATAGGGAGATACTCCCTCTGTCTTTCTTCTTCG	
3	pGEMT–T7 nested	Forward	TAATACGACTCACTATAGGGAGAAAGATACCAGGCGTTTCCCC	433
		Reverse	TAATACGACTCACTATAGGGAGAGCCGGATCAAGAGCTACCAA	
4	*Kr-h1* RT-PCR	Forward	TGAAGGTACATACCCGCACG	109
		Reverse	TAGTGGGCAACCTGATGCAA	
5	Elongation factor (Ef-1α)-RT-PCR	Forward	CGTTTACCGCTTCAGGACGT	91
		Reverse	GCATGCCTGGTTTCAGAATA	

Supplementary table S2: Summary of a three-way ANOVA analysis for Exp. 2 (Figure 2 B & C).

	Factor	F	DF	*P value*
Ovarian maturation	Treatment	6.689	3	***<0.001***
	Trial	0.856	1	*0.356*
	DMF vs. JH	10.266	1	***0.002***
	Treatment x Trial	0.814	3	*0.488*
	Treatment x DMF vs JH	0.099	3	*0.960*
	Trial x DMF vs JH	2.257	1	*0.135*
	Treatment x Trial x DMF vs JH	0.343	3	*p = 0.794*
Wax secretion	Treatment	16.646	3	***<0.001***
	Trial	2.692	1	*0.111*
	DMF vs. JH	2.040	1	*0.164*
	Treatment x Trial	1.289	3	*0.296*
	Treatment x DMF vs JH	0.282	3	*0.838*
	Trial x DMF vs JH	0.003	1	*0.953*
	Treatment x Trial x DMF vs JH	0.737	3	*p = 0.538*

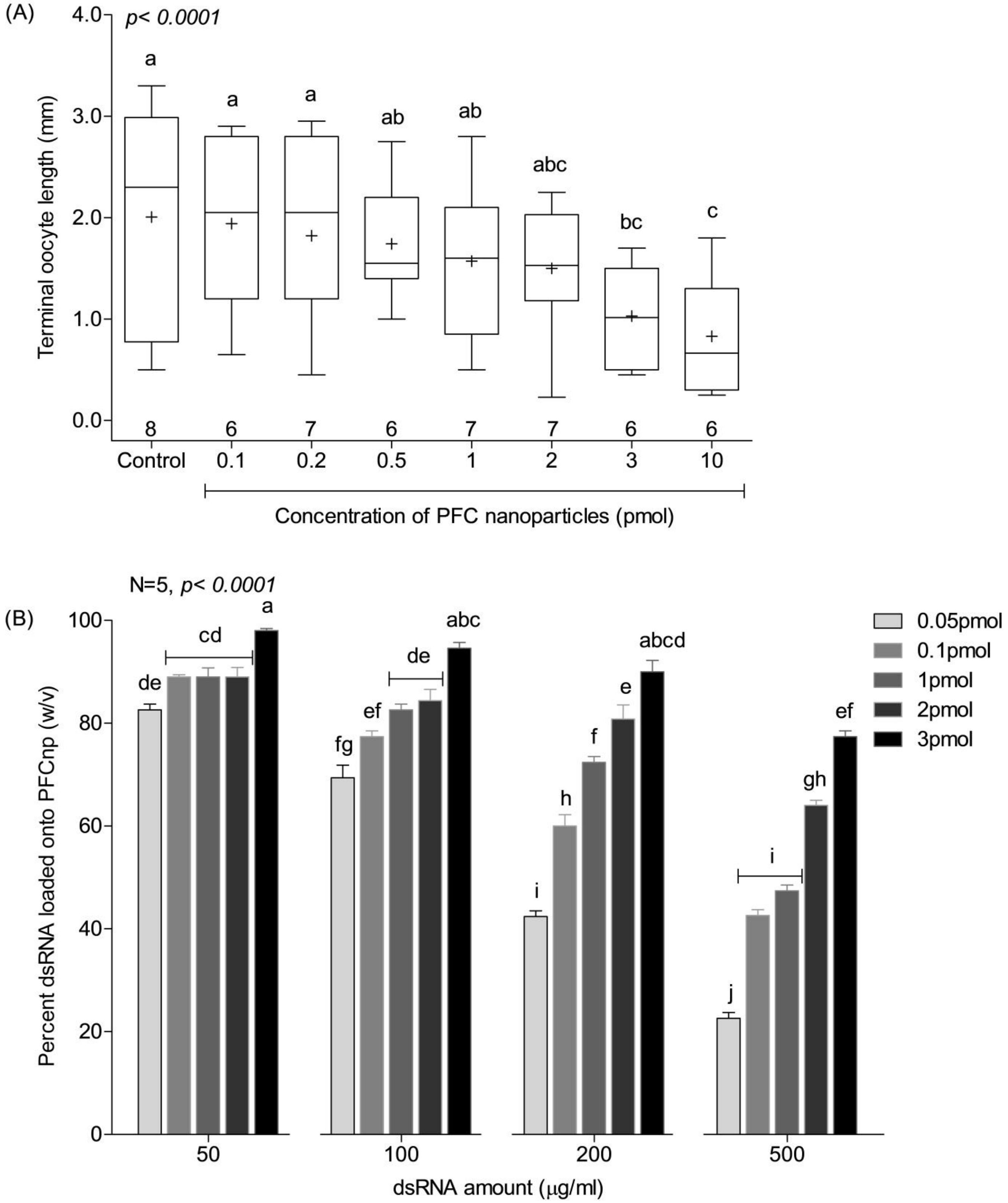

Supplementary Figure S1. The influence of PFCnp concentration on ovarian activity and dsRNA binding efficiency. (**A**) Ovarian activity and survival. The x-axis shows increasing concentration of PFCnp (not loaded with RNA) injected to bees. The number of bees surviving, out of a total of 8 injected bees, is shown in parentheses. Treatments with different letters are statistically different in a Kruskal-Wallis test followed by pairwise Dunn posthoc test. The box plots show ovarian activity at the age of 7 days. Each box plot shows the median (—), mean (+), and the box frame spans over the first to the third quartile. The whiskers depict the 5th/95th percentile; outliers are depicted with black dots. (**B**) dsRNA loading efficiency. The

darkness of the bar corresponds to the amount of PFCnp onto which the dsRNA was loaded. The bars and whiskers depict mean ± SE, N=5. Further details as in Figure 1. Bars marked with different small letters are significantly different in Two-way ANOVA and Bonferroni posthoc tests using log 10 transformed data.

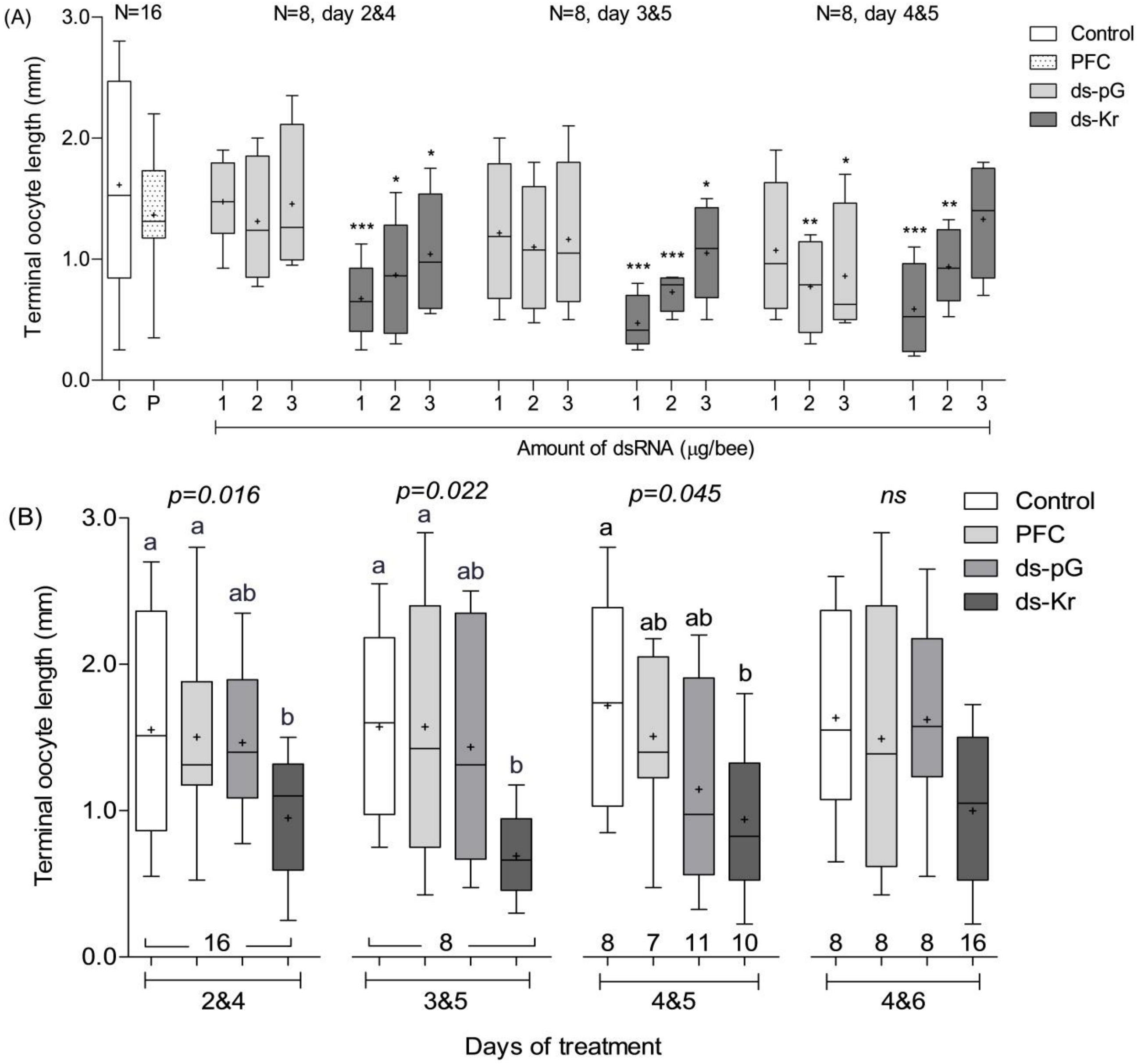

Supplementary Figure S2. The influence of the days of dsRNA injection on ovarian activity. (**A**) A summary of experiments in which we tested different amounts of dsRNA (1-3µg, as shown on the X axis). We loaded plasmid (ds-pG) or *Bombus terrestris Kr-h1* dsRNA (ds-Kr) onto 0.1pmol PFCnp. Ovarian activity was assessed on day 7 of the experiment. We compared each treatment group with the control group (C) using Unpaired t-test with Welch's correction for multiple comparisons. ** - α<0.05, ** - α<0.001, *** - α<0.0001*. (**B**) A summary of experiments in which we injected 1 µg control or *Kr-h1* dsRNA loaded to 0.1µg/µl PFCnp. Sample sizes is shown below each box plot. Treatments marked with different small letters are statistically different in a Kruskal-Wallis H Test, followed by Dunn's post-hoc analysis comparing each combination of injection days. For additional details, see Supplementary Figure S1.

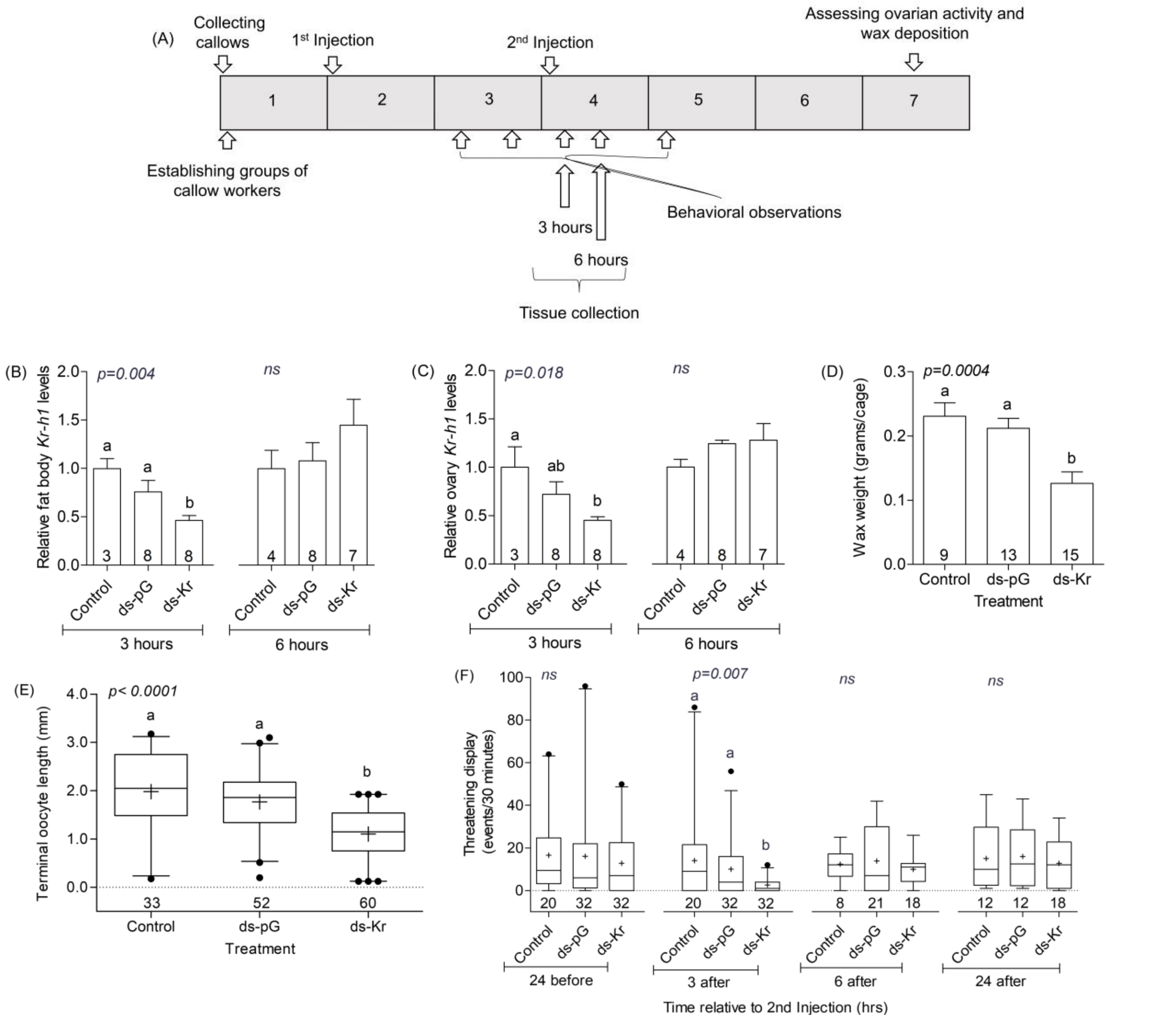

Supplementary figure S3: The influence of naked *Kr-h1* dsRNA injection on JH-regulated physiology and behavior. (A) general outline of the experiment. (B) Fat body *Kr-h1* mRNA levels. (C) Ovary *Kr-h1* mRNA levels. (D) Wax weight at the end of experiment. (E) Ovarian activity at 7 days of age. The vertical bars in panels B-D depict mean ± SE. (F) The amounts of threatening displays performed before and after the 2nd dsRNA injections. The details of the box plots in panel E are as described in Supplementary Figure S1. Treatments marked with different small letters are statistically different in either one-way ANOVA followed by Tukey post-hoc analysis for parametric data (B, C & D), or Kruskal Wallis H tests followed by Dunn's post-hoc analysis for non-parametric data (E & F). Numbers below the bars depict the sample size for each group; the sample size in D is the number of cages from which wax amount was measured.

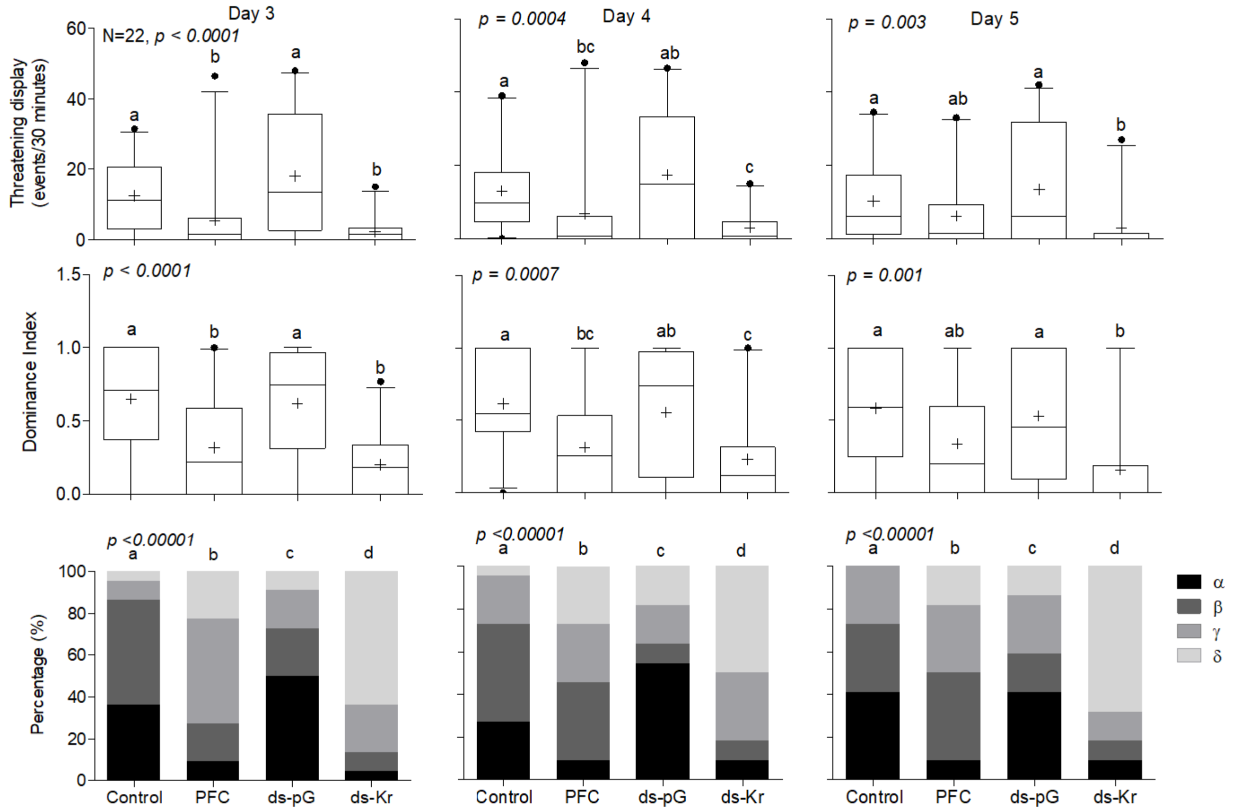

Supplementary Figure S4: The influence dsRNA mediated *Kr-h1* knock-down on dominance and agonistic behavior – separate analyses for each observation day. Top row: Threatening displays. Middle row: Dominance index. Lower row: Dominance rank. The right, central, and left columns summarize the observations on Day 3, 4, and 5, respectively. Other details as in Fig. 3.
